# Immunomodulatory effects of short-chain fatty acids and immune-supporting nutrients on slice cultures of head and neck tumors

**DOI:** 10.3389/fnut.2026.1731077

**Published:** 2026-03-13

**Authors:** Maria do Carmo Greier, Jozsef Dudas, Roland Hartl, Lukas Schmutzler, Benedikt Gabriel Hofauer

**Affiliations:** Department of Otorhinolaryngology, Head and Neck Surgery, Medical University of Innsbruck, Innsbruck, Austria

**Keywords:** immune modulation, immunometabolism, microbiota-derived metabolites, nutritional immunology, tumor metabolism

## Abstract

**Introduction:**

Head and neck squamous cell carcinoma (HNSCC) has a highly immunosuppressive tumor microenvironment (TME), which limits the effectiveness of conventional and immunotherapies. Metabolites derived from the gut microbiota, such as short-chain fatty acids (SCFAs), and targeted nutritional interventions, including immunonutrition (IN), have been proposed as ways of influencing tumor immunity and cell viability. However, the effects of these factors on the complex TME of HNSCC remain incompletely understood. Patient-derived organotypic slice cultures (SC) therefore provide a clinically relevant model to study these interactions.

**Methods:**

SC were generated from tumors of nine HNSCC patients and cultured under four conditions: control; SCFAs; IN (glutamine, alanine, and omega-3 fatty acids); and SCFAs combined with IN, for 4 days. Apoptotic activity was assessed via cleaved caspase-3 (CC3), and cytotoxic activity via Granzyme B (GrB) staining. Inflammatory markers (IL-1β, IL-6, TNFα and IFNγ) were quantified in cultured and treated tissue, as well as in the tissue’s supernatant. Quantitative immunohistochemistry (IHC) - based image analysis and dot blot assays were combined with statistical evaluation of patient- and treatment-specific effects.

**Results:**

Treatments with IN alone or in combination with SCFAs significantly reduced CC3 intensity, indicating decreased apoptosis. However, SCFA treatment alone increased CC3 intensity in SC of certain patients. GrB IHC intensity remained largely stable, with patient-specific differences driving the observed variability. Among the cytokines analyzed in the SC supernatants, TNFα and IL-1β were selectively modulated by IN and combined treatment, while IL-6 and IFN-*γ* remained largely unchanged. Analysis of cultured and treated tissue mirrored these trends, with TNFα and IFN-*γ* showing minimal variation and IL-6 being almost undetectable. These findings highlight significant heterogeneity in apoptotic and immune responses among patients.

**Conclusion:**

SCFAs and IN exert modest but selective effects on apoptosis and inflammatory pathways in HNSCC, whereas cytotoxic activity remains stable. These results support the potential of tailoring metabolic and nutritional interventions to individual patients to modulate the tumor immune microenvironment, and provide a rationale for integrating SCFAs and IN with immunotherapeutic strategies in HNSCC.

## 1 Introduction

Head and neck squamous cell carcinoma (HNSCC) is characterized by complex interactions within the tumor microenvironment (TME). Especially immune cells might display anti-tumor effects but also enable tumor survival and progression (1). HNSCC has several ways to escape the immune system. Tumor cells can evade immune recognition, resist cytotoxic attack, block immune cell activity, and attract suppressive cells like regulatory T cells tumor (2, 3). Certain cancer cell populations further contribute to this immunosuppressive environment by altering antigen presentation, secreting immunomodulatory cytokines, and driving therapy resistance (4). Together, these processes create a TME that strongly suppresses immune response against tumor cells and limits the success of both standard treatments and new immunotherapies (1). Study of these tumor-immune cell interactions requires tumor models that can reflect the complexity of human tumors. Standard cell cultures lack the original tissue structure, and animal models often fail to reproduce the human immune system. In contrast, patient-derived organotypic slice cultures (SC) have been developed as an advanced model that preserves tumor architecture, stromal support, and immune cell populations in conditions close to those found in patients (5). These cultures have been successfully used to study treatment responses, including the effects of oncolytic viruses and immunomodulatory agents, providing a versatile and clinically relevant model to investigate tumor–immune dynamics in HNSCC (6). While models like organotypic SC help us to study immune escape, it is clear that factors outside the tumor also play a role. In particular, metabolites of the gut and oral microbiota, are able to modulate inflammation, immunity, and even treatment outcomes in HNSCC (7).

Short-chain fatty acids (SCFAs), primarily acetate, propionate, and butyrate, are microbial metabolites generated through the fermentation of dietary fibers and play an important role in cancer biology (8). Recent studies have highlighted both anti- and pro-tumor genic activities depending on cancer type, concentration, and context. However, most studies suggest a strong immunomodulatory and anticancer potential (9, 10, 11, 12). SCFAs are able to regulate gene expression through epigenetic modulation like inhibition of histone deacetylases, promoting activation of tumor suppressor genes, induction of cell-cycle arrest and apoptosis in tumor cells (9, 10). Butyrate, in particular, has been shown to suppress oncogenic signaling pathways such as Wnt/*β*-catenin and PI3K/AKT while promoting mitochondrial apoptosis, highlighting direct cytotoxic effects on malignant cells (13). In addition to these tumor-intrinsic effects, SCFAs play a central role in regulating inflammation within the TME by modulating key cytokines such as IL-6, IL-1β, and TNF-*α*. Specifically, SCFAs can suppress pro-inflammatory cytokines like TNF-α and IL-6, while enhancing anti-inflammatory IL-10 and influencing IL-2 production, thereby limiting chronic inflammation and creating conditions that favor effective antitumor immune responses (14, 15). Still, their effects are concentration- and context-dependent, since in some settings SCFAs can also raise IL-6 and other inflammatory markers through pathways such as p38 MAPK, potentially promoting tumor progression (16, 17). This highlights their dual role anti-inflammatory and immunoregulatory metabolites (18, 19).

Beyond their direct effects on tumor cells, SCFAs can also shape the immune landscape within the TME. Butyrate and propionate for example, enhance CD8^+^ T cell metabolism and effector function by supporting mitochondrial fitness and promoting IFN-*γ* production, improving the efficacy of T cell therapy in preclinical models (20). Similarly, SCFAs have been shown to expand regulatory T cell populations via G-protein coupled receptors and epigenetic modulation (21, 22). This immunoregulatory effect can influence responsiveness to immunotherapy, as SCFA-mediated enhancement of effector T cells and natural killer cells has been associated with improved antitumor activity (18).

In the context of head and neck cancer, emerging evidence suggests that alterations in oral and gut microbiota, and their associated SCFA profiles, may directly affect tumor progression and immune interactions (23). Reduced SCFA levels in head and neck cancers were correlated with microbial dysbiosis, potentially contributing to a more immunosuppressive microenvironment (7, 24). Supplementation or modulation of SCFA-producing bacteria has been proposed as a therapeutic strategy to restore immune function and sensitize tumors to immunotherapy (9, 22). Therefore, SCFAs are seen as critical mediators linking diet, microbiota, and cancer immunity, with significant potential for application in HNSCC.

The interaction between cancer cells and the immune system influences both, tumor progression and therapeutic outcomes. The immunosuppressive TME of HNSCC is characterized by T cell exhaustion, presence of immunosuppressive cells, and upregulation of checkpoint molecules such as PD-1 and PD-L1, which contribute to limited efficacy of conventional therapies (2). The KEYNOTE-040 trial for example showed that the immune checkpoint inhibitor antibody pembrolizumab can improve survival in patients with recurrent or metastatic HNSCC compared to standard chemotherapy. However, only a small number of patients have long-lasting benefits, showing the need for new ways to strengthen the immune response against tumors (25). Other treatment strategies like radiotherapy, can both help and harm the immune system. Positive effects are triggering cancer cell death and release of new antigens. Negative effect is the weakening of immunity by changing the TME (26). Chemoradiation is also widely used and effective, but it often causes strong side effects and does not fully restore long-term immune function (27).

In this context, immunonutrition may support antitumor immunity and improve treatment tolerance. Nutrients such as glutamine, omega-3 fatty acids, and nucleotides have been shown to modulate immune responses by enhancing T cell proliferation, restoring macrophage activity, and reducing systemic inflammation (28). Glutamine, in particular, is essential for rapidly proliferating immune cells and plays a central role in supporting lymphocyte activity during stress and cancer therapy (29, 30). Omega-3 fatty acids, on the other hand, exert anti-inflammatory and immunomodulatory effects, linked to reductions in cachexia, improved muscle preservation, and enhanced chemotherapy sensitivity in several cancers (31, 32). In HNSCC and other solid tumors, omega-3 enriched diets have been associated with reduced systemic inflammation and improved treatment tolerance, further supporting their potential role as adjuncts in cancer care (29). Clinical studies in HNSCC patients undergoing surgery, demonstrated that perioperative immune-enhancing diets reduced infectious complications and hospital stay (33, 34). Furthermore, studies also support that immunonutrition improves nutritional status, reduces treatment-related toxicities, and may enhance overall survival in HNSCC (33, 35). Importantly, in patients receiving chemoradiation, immunonutrition was associated with improved treatment compliance and reduced mucositis, suggesting that it can be used with cytotoxic therapy while reducing adverse effects (36). Not only nutrient supplementation, but also modulation of the microbiota and microbiota-derived metabolites is a possible way to increase immune function during cancer therapy (37).

Integrating these microbiota-mediated pathways with immunonutrition may therefore offer a strategy, that supports the host immune system while reshaping the TME to favor immune checkpoint inhibitor efficacy and improved responses to chemo- and radiotherapy. Such combined interventions hold promise for overcoming immune resistance in HNSCC and improving patient outcomes. Therefore, the main aim of this study was to use patient-derived tumor SC to investigate how SCFAs, alone or combined with immunonutrition, affect the immune landscape in HNSCC, through analysis of cytotoxic activity and measurement of inflammatory cytokines.

## 2 Methods

### 2.1 Study population and preparation of HNSCC slice cultures

Patients with newly diagnosed, locally advanced HNSCC treated at the Department of Otorhinolaryngology – Head and Neck Surgery, Medical University of Innsbruck, between November 2024 and June 2025 were enrolled. Eligible participants were adults over 18 who underwent diagnostic endoscopy with anesthesia and biopsy and had a T3-T4 primary tumor. Patients were excluded if anesthetized endoscopy was contraindicated or if histopathology showed a non-HNSCC tumor. The study was approved by the Ethics Committee of the Medical University of Innsbruck (Approval number: 1199/2019), and all patients provided written informed consent. Biopsies were taken from 9 patients from non-necrotic tumor regions using biopsy forceps and immediately processed for SC, which were cut into 250-μm sections using a Compresstome® VF-310-0Z (Precisionary Instruments LLC, Ashland, MA, USA).

### 2.2 Culture conditions

SC were placed in 24-well plates (Corning, Durham, USA) and maintained under four different conditions. These conditions were control, SCFAs, immunonutrition (IN) and SCFAs + immunonutrition (SCFA+IN). Control condition contained 1 mL of serum-free keratinocyte medium (Keratinocyte SFM; #10724-011, Thermo Fisher, Waltham, MA) supplemented with Antibiotic-Antimycotic (#15240062, Thermo Fisher, Rochester, NY, USA) at 1:100, providing final concentrations of 100 μg/mL streptomycin, 250 ng/mL amphotericin B, and 100 units/mL penicillin. For SCFA condition, 170 μL of SCFA-mix was added to 1 mL of serum-free keratinocyte medium. This SCFA-mix was prepared using the short chain fatty acids product of (#SBR00030, Sigma-Aldrich, Darmstadt, Germany), We fully considered the treatment concentration suggestions of the provider Sigma-Aldrich: sodium Acetate: 70 mM, sodium propionate: 30 mM, sodium butyrate: 20 mM. We prepared the solutions in the following way:

From 1 M of sodium acetate 7 mL was added, from 0.5 M of sodium propionate 6 mL was added and from 0.5 M of sodium butyrate 4 mL was added and these were mixed. Final concentrations were: 60 mM sodium acetate; 25.64 mM sodium proprionate; 17.09 mM sodium butyrate. These concentrations are comparable with published treatments (38).

For IN condition, the clinically used IN treatment was adapted to the SC conditions in our experimental work. In the cultivation medium IN was mixed containing 30 mL of serum-free keratinocyte medium, 10 mL of Dipeptiven (#11051014, Fresnius Kabi, Bad Homburg, Germany), 10 mL of Omegaven (#11110004, Fresnius Kabi, Bad Homburg, Germany) and 1 mL of amphotericin B (#15290–026, Thermo Fisher, Paisley, UK). For SCFA+IN, 170 μL of SCFA-mix was added to the 1 mL of immunonutrition medium. SC were kept in a 37 °C incubator (5% CO2) for a total of 4 days.

### 2.3 Collection of supernatant and dot blot analysis

After culture, SCs were fixed and the supernatants from each well were collected. IL-1β (1:1000;#12703S, Cell signalling), IL-6 (1:500, # MAB206, RnD systems, Minneapolis, USA), IFN-*γ* (1:500, #ab231036, Abcam), Granzyme B (GrB; 1:200, #3002-MSM4-P1, Invitrogen, Massachusetts, USA) and TNFα (1:500, #MAB610, RnD systems) levels were assessed by the aforementioned antibodies and dilutions using a dot blot approach. Circles were lightly drawn with a pencil on Amersham Protran 0.2 μm nitrocellulose membranes (#10600001, GE Healthcare, UK) to mark spots for each supernatant. Membranes were treated with 10% methanol containing blotting solution (Invitrogen, Darmstadt, Germany) and air-dried. From each sample, 20 μL of supernatant was applied to the marked areas. After drying, membranes were rehydrated in TBS and blocked for one hour at room temperature using Invitrogen TBS Starting Blocking Solution (#37542, Invitrogen). Primary antibody incubation was performed overnight with IL-1, IL-6, IFN-*γ*, Granzyme B (GrB) and TNFα specific antibodies diluted as mentioned above, in blocking solution containing 0.2% Tween 20. Detection was carried out with anti-mouse IgG IR 800 (1:2500; #AC2135, Azure; Houston, TX, USA), or anti-rabbit IgG 800CW (1:5000, LiCor, Houston, TX, USA). After background correction, optical densities of the dots were quantified using ImageJ 1.46r (NIH, USA), with background signals subtracted for each measurement as published before (39).

### 2.4 Staining procedure

Following the culture period, SCs were fixed in 4% paraformaldehyde (#FN-10000-4-1, SAV Liquid Production, Germany) and rinsed with phosphate-buffered saline (PBS; Fresenius Kabi, Germany) after overnight fixation. Fixed tissues were embedded in paraffin using the Histos 5 microwave system (Milestone, Italy), and 5-μm sections were cut with a Microm microtome (Heidelberg, Germany) and dewaxed. Hematoxylin-eosin staining was performed according to Gill following the manufacturer’s instructions (Hematoxylin: #1.05174.0500; Eosin: #1098441000 Merck, Germany). Immunostaining for cleaved caspase-3 (CC3) was conducted on a Ventana Discovery Ultra immunostainer (Ventana Roche, USA) using a polyclonal rabbit CC3 antibody (diluted at 1:400, #9661, Cell Signalling Technology). GrB was detected with a mouse monoclonal IgG2b antibody (diluted at 1:200, #3002-MSM4-P1, Invitrogen, Massachusetts, USA). IL6 was detected with mouse monoclonal IgG1 antibody (diluted at 1:30, # MAB206, RnD systems, Minneapolis, USA), IFN-*γ* with rabbit monoclonal IgG (diluted at 1:500, #ab231036, Abcam, Cambridgeshire, UK) and TNFα with rabbit monoclonal IgG antibody (diluted at 1:30, #MAB610, RnD systems, Minneapolis, USA). Detection of primary antibodies was performed with a Ventana universal secondary antibody (#05268877) and visualized using the Ventana DAB Map Detection Kit (#05266360001, Roche, Mannheim, Germany). Nuclei were counterstained with Hematoxylin II (#5277965001, Roche, Mannheim, Germany), after which slides were dehydrated, mounted with Entellan (Merck, Darmstadt, Germany), and left to dry overnight.

### 2.5 Image acquisition

Brightfield images were captured using a TissueFAXS PLUS system (TissueGnostics GmbH, Vienna, Austria) coupled to a Zeiss Axio Imager Z2 microscope (Jena, Germany) with a 20×, 0.6 NA apochromat air objective. For this, a colour Pixelink Camera PL-D674CU-CYL (Ottawa, Canada) was used. All definable tissue regions were captured using the TissueFAXS 7.1 software (TissueGnostics), enabling image cytometry analysis of individual regions. Because of intra-sample heterogeneity, results were analyzed per tissue region rather than averaged across the entire sample. Quantification of staining in defined tissue regions was performed using HistoQuest 7.1 software (TissueGnostics GmbH, Vienna, Austria). Images were first imported into the software, and regions unsuitable for analysis, such as necrotic areas, were excluded. Cells were identified based on nuclear counterstaining with hematoxylin, and IHC signals were assigned to corresponding nuclei. Nuclear segmentation followed protocols described in our previous work (39). Mean intensity values represented the color intensity of the IHC signal, with outputs including total cell counts, sample area, and mean marker intensity at 8-bit resolution. All intensity measurements were recorded as obtained, without adjustment, and only included events associated with a detectable nucleus; nonspecific background signals were excluded.

### 2.6 Data analysis

Data were collected for all stained regions and were evaluated at the region level. Based on the distribution of the individual values, means or medians were used for further comparisons. Normal or non-parametric distribution of the individual values were first identified. For comparison of means, unpaired t test with Welch’s correction, or for more conditions compared Kruskal-Wallis-test were used. The confidence interval was 95%, and statistical significance was claimed at *p* < 0.05. To evaluate whether patient-specific factors or experimental conditions had an effect on measured outcomes, univariate analysis was performed. All calculations were performed with SPPS Statistics Ver. 30 (IBM, Armonk, NY). Estimated marginal means (EMM) and their standard errors (SEM) were graphically presented using GraphPad Prism 10 (GraphPad Software, San Diego, CA, USA). All raw data are and statistic files loaded up to 10.5281/zenodo.

## 3 Results

Overall, tumors of 9 patients have been used to create SC. Of these, three tumors were located in the oropharynx, five in the oral cavity and one in the larynx. From 9 tumors, three were p16 positive and the rest was p16 negative. Patient ages ranged from 39 to 78 years (average age 67.7), from which one patient was female and eight were male. HPV background based on p16 IHC and HPV PCR were obtained from routine pathological diagnostics (Table 1).

**Table 1:** Clinical and molecular characteristics of patients with HNSCC.

Patient	Sex	Age	Location	T stage	p16	CD45	p53
1	M	72	Oropharynx	T4a	Negative	1	1
2	M	61	Larynx	T3	Negative	3	3
3	F	72	Oral cavity	T4b	Positive	3	3
4	M	75	Oral cavity	T2	Negative	1	3
5	M	74	Oral cavity	T2	Positive	2	1
6	M	78	Oropharynx	T4a	Negative	3	2
7	M	69	Oral cavity	T4a	Negative	3	3
8	M	39	Oral cavity	T4a	Negative	1	1
9	M	69	Oropharynx	T2	Positive	3	1

CD45 scoring: 1 = immune desert, 2 = excluded, 3 = infiltrated. p53 scoring: 1 = wildtype, 2 = protein stabilization, 3 = mutated.

### 3.1 Impact of SCFAs and immunonutrition on apoptosis in SC

**Figure 1:**
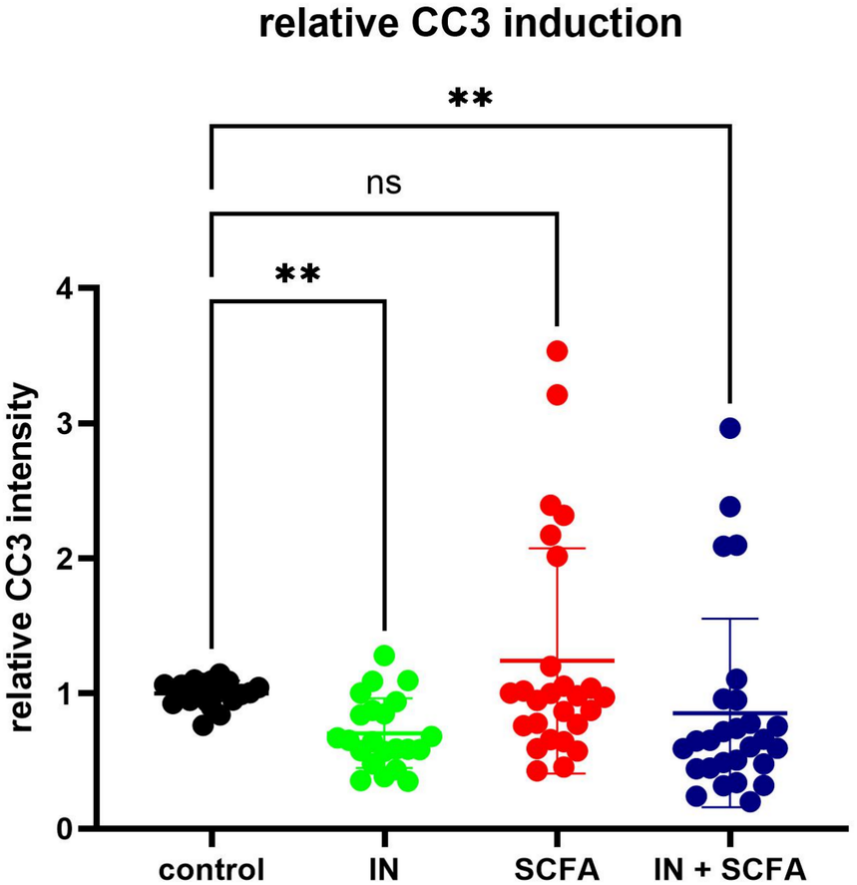
Means of CC3 intensity in four different culturing conditions (ns, not significant; **p < 0.01). Means were compared by Kruskal-Wallis test.

To gain an initial overview of the impact of SCFAs and IN on apoptosis within SC, the median relative CC3 intensity was evaluated across the four experimental conditions. Compared to control conditions, IN alone (*p* = 0.0018) and IN combined with SCFAs (*p* = 0.001) were associated with a significantly lower apoptotic rate, whereas SCFAs alone did not show significant changes in CC3 intensity relative to control (*p* = 0.999; Figure 1).

To evaluate whether patient-specific factors or experimental conditions had an effect on relative CC3 intensity, an univariate analysis was performed. The analysis included all available stained tissue regions, stratified by patient (*n* = 9) and by treatment conditions (Table 2). In total, between 6 and 14 regions were analyzed per patient, and between 20 and 27 regions per treatment condition. Results indicated that patient identity had a significant effect on relative CC3 intensity (*p* < 0.001), which indicates inter-patient heterogeneity in apoptotic activity within SC. Treatment conditions were also identified as a significant determinant (*p* < 0.001), which suggests nutritional and metabolic influences on apoptosis. Moreover, the combination of patient and condition was also significant (*p* < 0.001), indicating that the effect of the treatments was not the same across all patients but modulated by patient-specific tumor characteristics.

**Table 2:** Overview of stained tissue regions per patient and treatment condition.

Patient	*N*	Treatment condition	*N*
1	12	Control	20
2	12	Immunonutrition	22
3	10	SCFAs	26
4	6	Immunonutrition + SCFAs	27
5	12		
6	14		
7	11		
8	11		
9	7		

The table displays the number of analyzed regions (N) per patient (1–9) and per experimental conditions.

**Figure 2:**
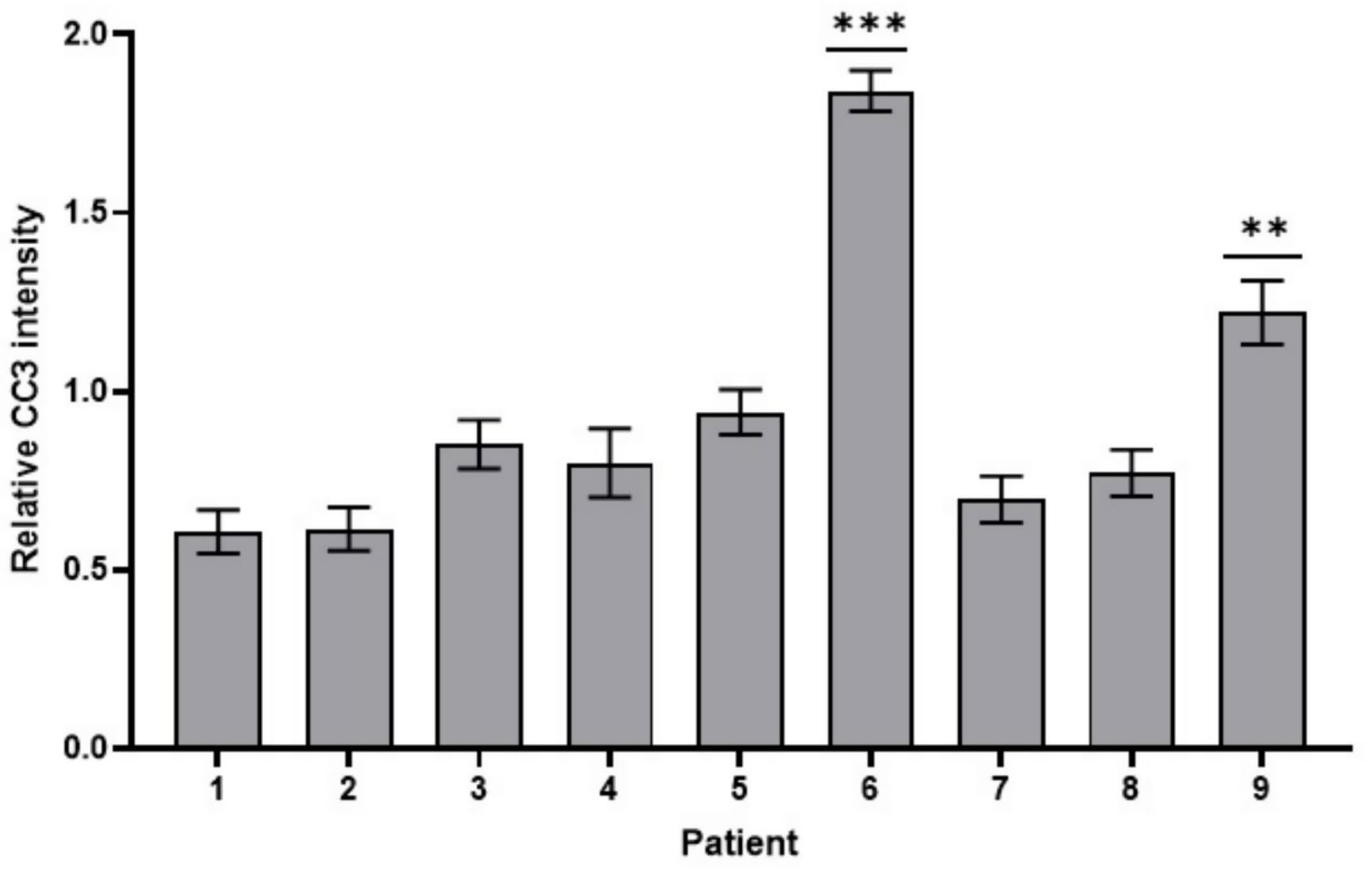
EMM of relative CC3 intensity in 9 patients. Patient 6 and patient 9 (highest intensities) significantly differed from all other patients (Error bars: SEM; **p < 0.01; ***p < 0.001).

To assess the influence of patient specific differences on relative CC3 intensity, estimated marginal means (EMMs) were calculated for all nine patients (Figure 2). The analysis revealed variability across patients, confirming the significant patient effect (*p* < 0.001). The F test value of the effect of parameter “patient” was: 42.844.

Patients 1 and 2 showed the lowest mean relative intensities and patients 7 and 8 also demonstrated comparatively low relative intensities. Patients 3, 4, and 5 exhibited intermediate levels of CC3 activity. In contrast, patients 6 and 9 showed the highest relative CC3 intensities and significantly differed from all other patients (*p* < 0.001; *p* < 0.01, respectively). Patient 6 in particular showed an elevated mean value of 1.842 ± 0.057 (95% CI: 1.728–1.956), which was more than twice as high as the intensities observed in most other patients. Patient 9 also demonstrated increase CC3 activity, with a mean of 1.221 ± 0.089 (95% CI: 1.043–1.398).

**Figure 3:**
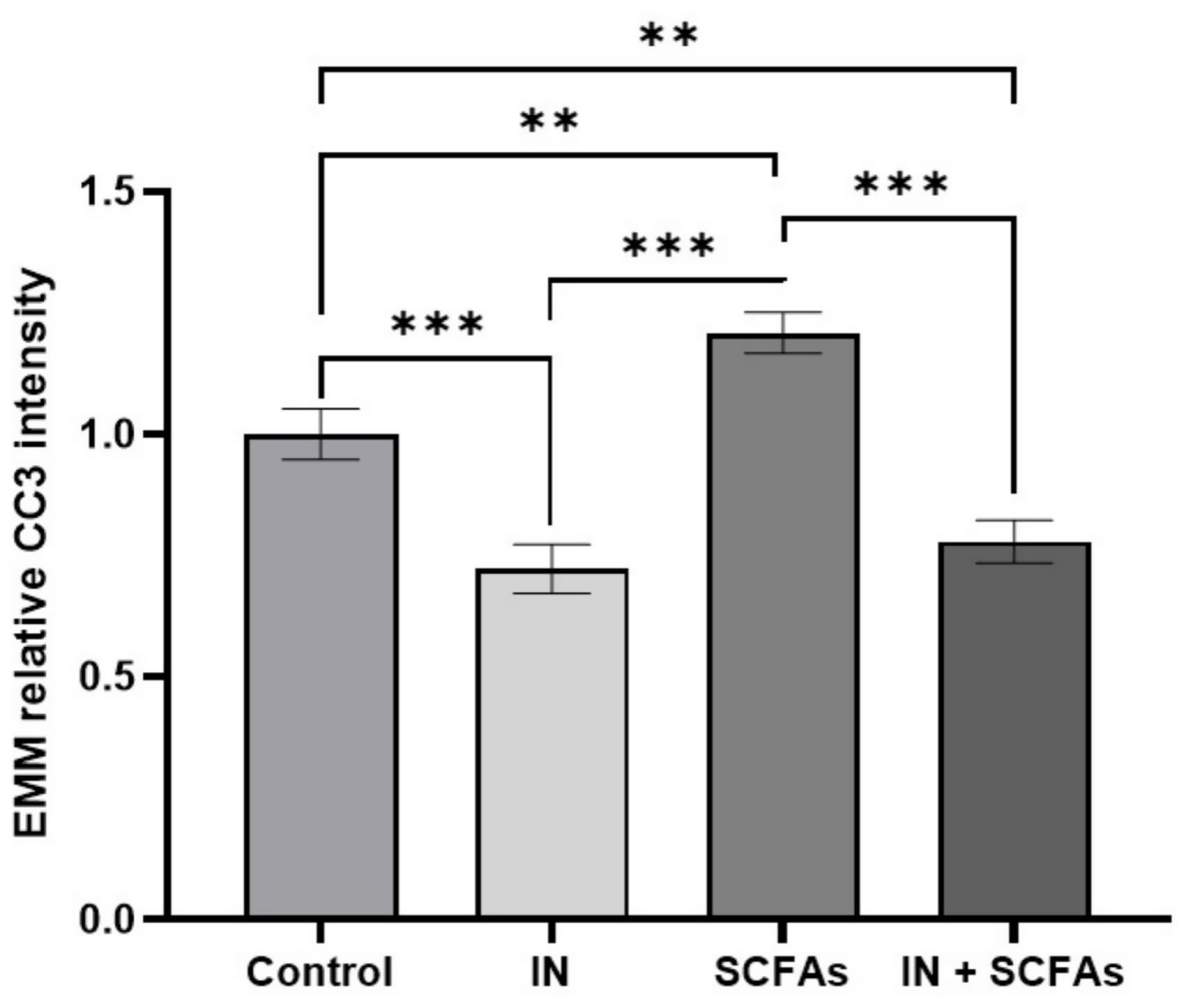
Estimated marginal means (EMMs) of relative CC3 intensity across treatment conditions control, immunonutrition (IN), short-chain fatty acids (SCFAs), and combined IN + SCFAs (Error bars: SEM; **p < 0.01, and ***p < 0.001).

To see how the four experimental treatments influenced relative CC3 intensity, EMMs were calculated and analyzed (Figure 3). The univariate analysis revealed a significant overall effect of treatment condition (*p* < 0.001), confirming that apoptotic activity varied according to the applied intervention. The control group showed a mean relative intensity of 1.000 ± 0.052 (95% CI: 0.897–1.103). Compared to control, IN led to a reduction (0.723 ± 0.050, 95% CI: 0.623–0.822), while IN and SCFAs showed a slightly higher but still suppressed level (0.779 ± 0.044, 95% CI: 0.692–0.867). In contrast, SCFA treatment alone resulted in an increase (1.209 ± 0.042, 95% CI: 1.124–1.294), exceeding the control mean.

Pairwise comparisons confirmed several significant differences (Figure 3). Relative to control, IN produced a significant decrease (mean difference = 0.277, *p* < 0.001), and IN + SCFAs was also lower (mean difference = 0.221, *p* = 0.002). The F test value of the effect of parameter “treatment conditions” was: 24.726.

Conversely, SCFAs induced a significant increase compared to control (mean difference = −0.209, *p* = 0.003). Direct comparisons between treatments highlighted the strongest contrast between SCFAs and IN, where SCFAs yielded significantly higher CC3 intensity (mean difference = 0.486, *p* < 0.001). SCFAs also exceeded IN + SCFAs (mean difference = 0.429, *p* < 0.001). Additionally, IN remained lower than IN + SCFAs (mean difference = −0.057, *p* = 0.397), which was not significant.

The treatment-based effects were less strong than the patients-based differences.

**Figure 4:**
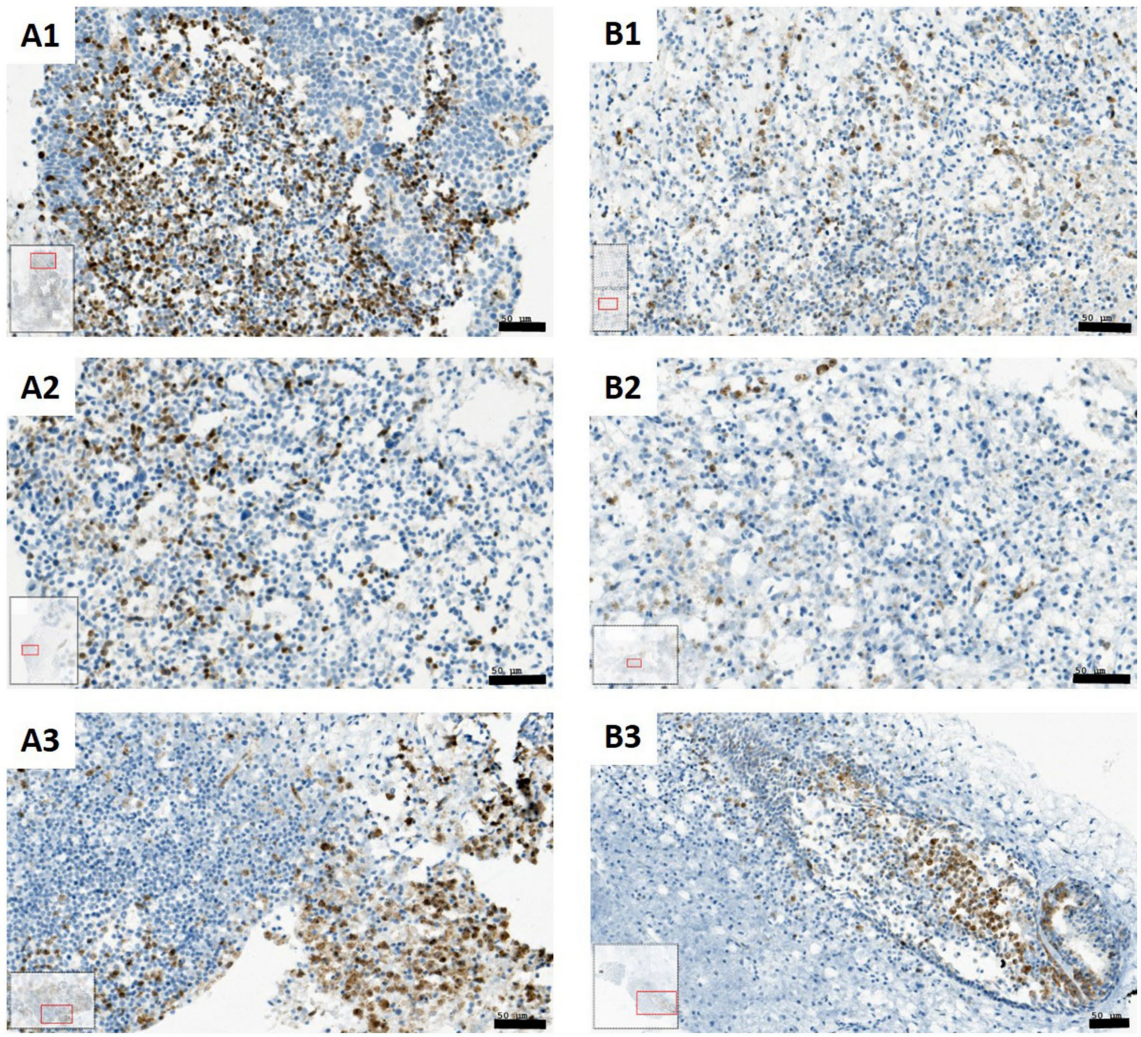
CC3 staining comparing control condition with immunonutrition and immunonutrition combined with SCFAs (blue: CC3 negative cells; brown: CC3 positive cells). **(A1)** Control condition patient 3; CC3 positive cells in stroma and tumor regions. **(A2)** Immunonutrition patient 3; lower amount of CC3 positive cells compared to control. **(A3)** SCFA condition patient 3; CC3 positive cells mostly in tumor regions. **(B1)** Control condition patient 6; CC3 positive cells in stroma and tumor regions. **(B2)** Immunonutrition patient 6; lower amount of CC3 positive cells compared to control. **(B3)** Immunonutrition + SCFAs patient 6; CC3 positive cells mostly in tumor regions (Scale bars: 50 μm).

In addition to the quantitative analyses of relative CC3 intensity, visual inspection of IHC images from representative patients was performed, which showed reductions in CC3-positive cells under IN and IN + SCFAs conditions (Figure 4). In SC-sample of patient 3, control tissue (A1) strong CC3 staining in both tumor and stromal regions was observed. IN alone (A2) visibly reduced the number of positive cells, whereas SCFAs alone (A3) seemed to retain a pattern similar to control. The combination of IN and SCFAs reduced CC3-positive cells overall and seemed to concentrate staining mainly in tumor areas. A similar trend was observed in patient 6. The control sample (B1) showed staining in stroma and tumor areas, IN alone (B2) reduced the number of positive cells, and IN+SCFAs (B3) further diminished CC3 staining with apparent tumor-focused localization. These visual observations are in line with the statistical analyses, showing that IN, alone or with SCFAs, reduces overall apoptotic activity. In addition, SCFAs or the combination of IN and SCFAs seems to focus CC3 reaction mainly to tumor areas.

The interaction between patient and treatment condition also showed a clear and statistically significant influence on relative CC3 intensity (*p* < 0.001). EMM for each patient and condition are summarized in Table 3. While the control condition was normalized to 1.0 across all patients, different conditions caused changes that differed in size and direction. In samples of several patients, IN or the combined IN + SCFA treatment was associated with a clear reduction in relative intensity compared with control. For example, Patient 1 showed a decrease under both IN (0.57) and IN + SCFAs (0.37) and a similar pattern was observed in Patient 2. In contrast, certain patients exhibited the opposite trend. Patient 6 demonstrated a strong induction of CC3 activity, with SCFAs alone producing the highest mean value observed (2.83; 95% CI 2.62–3.04) and the combination with IN remaining elevated (2.38; 95% CI 2.17–2.60). Patient 9 also displayed an increase with SCFAs (2.17; 95% CI 1.87–2.47), despite only minimal changes with IN. Other patients, such as Patient 5, showed slight alterations under any treatment, which may indicate a more neutral response.

**Table 3:** Estimated marginal means (EMMs) of relative CC3 intensity for each patient across all treatment conditions.

Patient	Condition	EMM	SEM	95% Confidence interval	
				Lower bound	Upper bound
1	Control	1.000	0.122	0.756	1.244
	IN	0.569	0.122	0.325	0.813
	SCFAs	0.492	0.122	0.248	0.736
	IN + SCFAs	0.368	0.122	0.124	0.612
2	Control	1.000	0.122	0.756	1.244
	IN	0.363	0.122	0.119	0.607
	SCFAs	0.770	0.122	0.526	1.014
	IN + SCFAs	0.326	0.122	0.082	0.570
3	Control	1.000	0.149	0.701	1.299
	IN	0.643	0.122	0.399	0.887
	SCFAs	1.028	0.149	0.729	1.327
	IN + SCFAs	0.738	0.122	0.494	0.982
4	Control	1.000	0.211	0.577	1.423
	IN	0.585	0.211	0.162	1.007
	SCFAs	1.026	0.122	0.782	1.270
	IN + SCFAs	0.591	0.211	0.169	1.014
5	Control	1.000	0.122	0.756	1.244
	IN	0.925	0.149	0.627	1.224
	SCFAs	1.002	0.122	0.758	1.246
	IN + SCFAs	0.841	0.106	0.630	1.053
6	Control	1.000	0.122	0.756	1.244
	IN	1.156	0.122	0.912	1.400
	SCFAs	2.828	0.106	2.617	3.039
	IN + SCFAs	2.383	0.106	2.172	2.595
7	Control	1.000	0.149	0.701	1.299
	IN	0.697	0.122	0.453	0.941
	SCFAs	0.625	0.122	0.381	0.869
	IN + SCFAs	0.471	0.122	0.227	0.714
8	Control	1.000	0.149	0.701	1.299
	IN	0.627	0.122	0.383	0.871
	SCFAs	0.941	0.122	0.697	1.185
	IN + SCFAs	0.521	0.122	0.277	0.765
9	Control	1.000	0.211	0.577	1.423
	IN	0.939	0.211	0.516	1.361
	SCFAs	2.168	0.149	1.869	2.467
	IN + SCFAs	0.775	0.122	0.531	1.019

Values represent mean CC3 intensity ± SEM with corresponding 95% confidence intervals.

These results show inter-individual heterogeneity in apoptotic responses to nutritional interventions. Whereas some tumors were highly sensitive to SCFA exposure, others were largely unaffected or even showed reduced activity with combined treatments.

### 3.2 Effect of SCFAs and immunonutrition on Granzyme B activity

**Figure 5:**
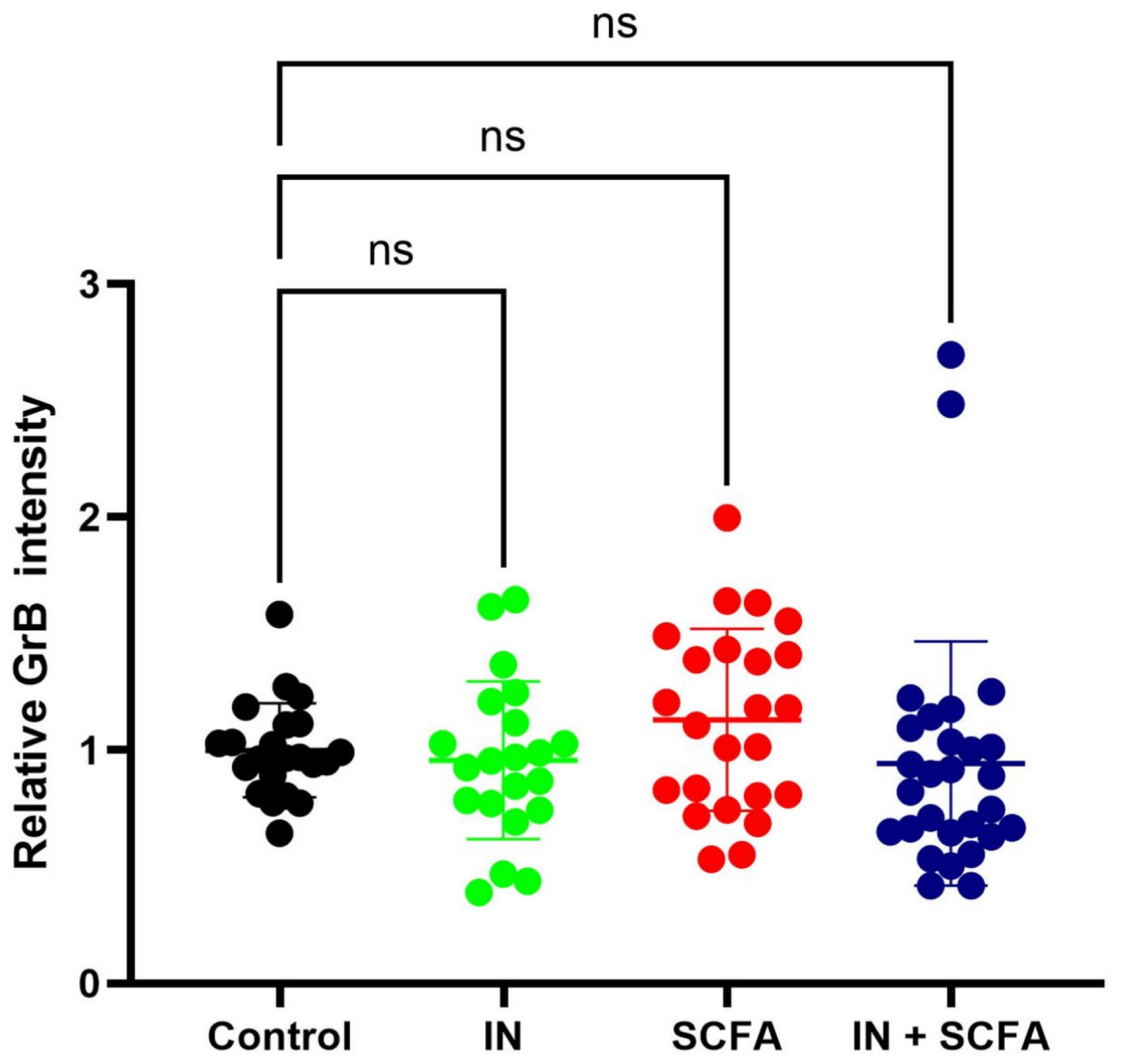
Means of relative GrB intensity in four different culturing conditions (ns, not significant).

To evaluate the impact of SCFAs and IN on cytotoxic T cell activity within SC, relative GrB intensity was analyzed across the four experimental conditions. The mean GrB intensity revealed no apparent differences compared to control, indicating that neither SCFAs, IN, nor their combination significantly modulated GrB immunohistochemical intensity (Figure 5).

To analyze the effects of patient-specific factors and experimental conditions on GrB reaction, an univariate analysis was performed across all available tissue regions. The analysis included nine patients, with 3–14 regions per individual, and four treatment conditions, with 21–28 regions per condition (Table 4).

**Table 4:** Overview of stained tissue regions per patient and treatment condition.

Patient	*N*	Condition	*N*
1	11	Control	22
2	12	Immunonutrition	21
3	11	SCFAs	24
4	3	Immunonutrition + SCFAs	28
5	10		
6	14		
7	12		
8	11		
9	11		

The table displays the number of analyzed regions (N) per patient (1–9) and per experimental conditions.

Patients-related differences had a significant strong influence on GrB levels with high F-test value (*p* < 0.001; *F* = 22.746), indicating heterogeneity in immune activity between patients. Treatment condition also showed a significant, but weak effect (*p* = 0.03; *F* = 3.192) with low effect size, suggesting that nutritional and metabolic interventions may moderately modulate GrB levels. The interaction between patient and treatment effects was significant (*p* < 0.001; *F* = 5.149), demonstrating that the impact of experimental conditions varied across patients and was influenced by individual tumor characteristics. A further parameter, namely the infiltration of CD45^+^ cells seemed to influence both the effects of SCFAs and IN, but the interaction effects were not significant. Tendentially reduction of GrB intensity after IN treatment was more visible in samples with immune desert, and the GrB activation effect of SCFAs was more pronounced in samples with immune infiltrate (Supplementary Table 1). This finding allows a blink into the patients-related conditions, which influence the outcome of treatments.

**Figure 6:**
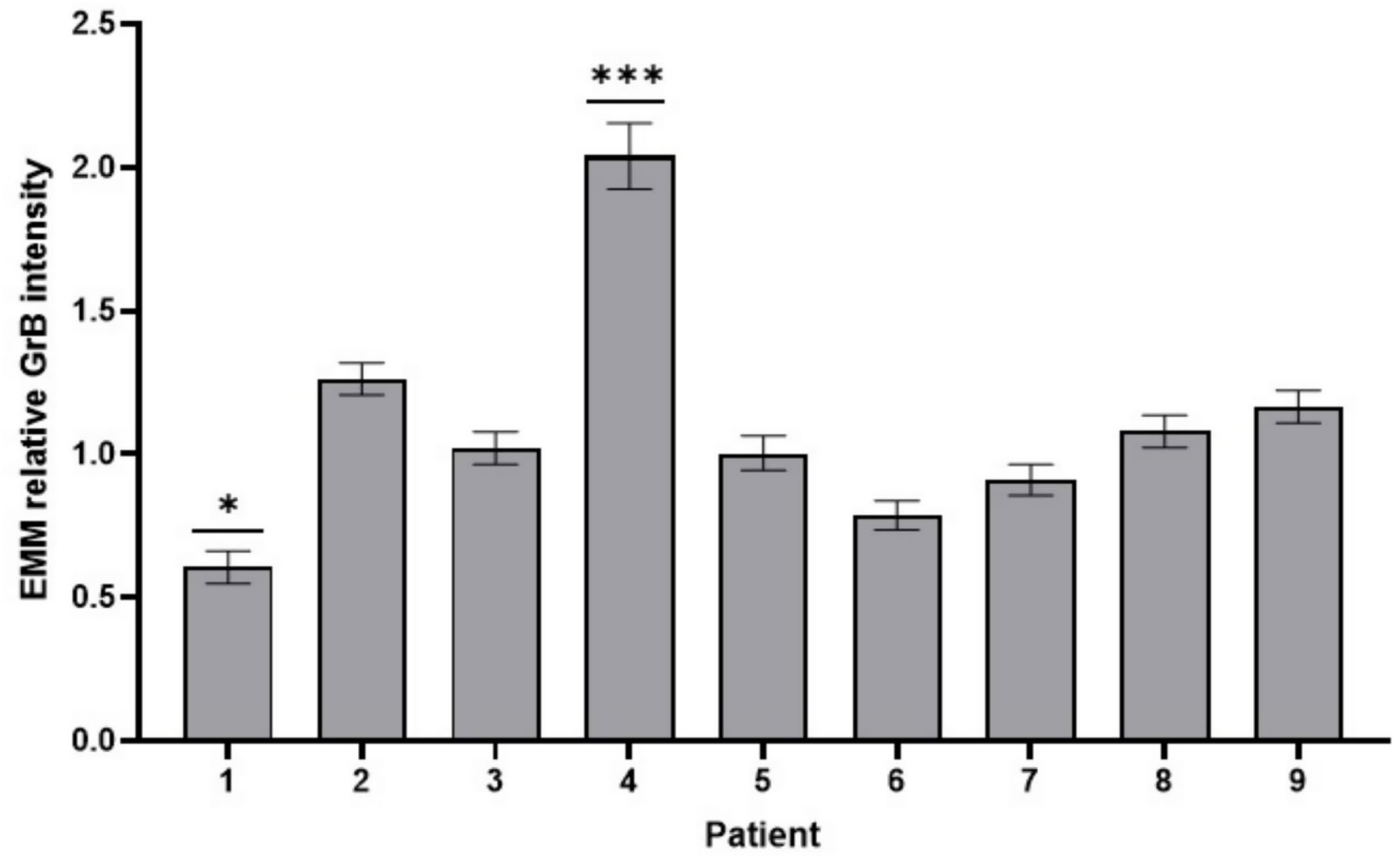
EMM of relative GrB intensity in 9 patients. GrB relative intensity in tissue cultures of Patient 1 (lowest intensity) and patient 4 (highest intensity) significantly differed from those of all other patients (Error bars: SEM; *p < 0.05; ***p < 0.001).

To analyze inter-individual variability in relative GrB intensity, EMMs were calculated for all nine patients (Figure 6). The results indicated differences between patients, in line with the significant patient effect observed in the overall analysis. Patient 1 showed the lowest relative GrB intensity with a mean of 0.606 ± 0.057 (95% CI: 0.491–0.720), while patient 4 exhibited the highest value at 2.040 ± 0.115 (95% CI: 1.810–2.269). Intermediate levels were observed in the remaining patients samples. Pairwise comparisons revealed that tissue samples of patient 1 and patient 4 differed significantly from all other individuals (patient 1: *p* < 0.05 and patient 4: *p* < 0.001). These findings indicate that patient-specific factors strongly influence GrB intensity.

**Figure 7:**
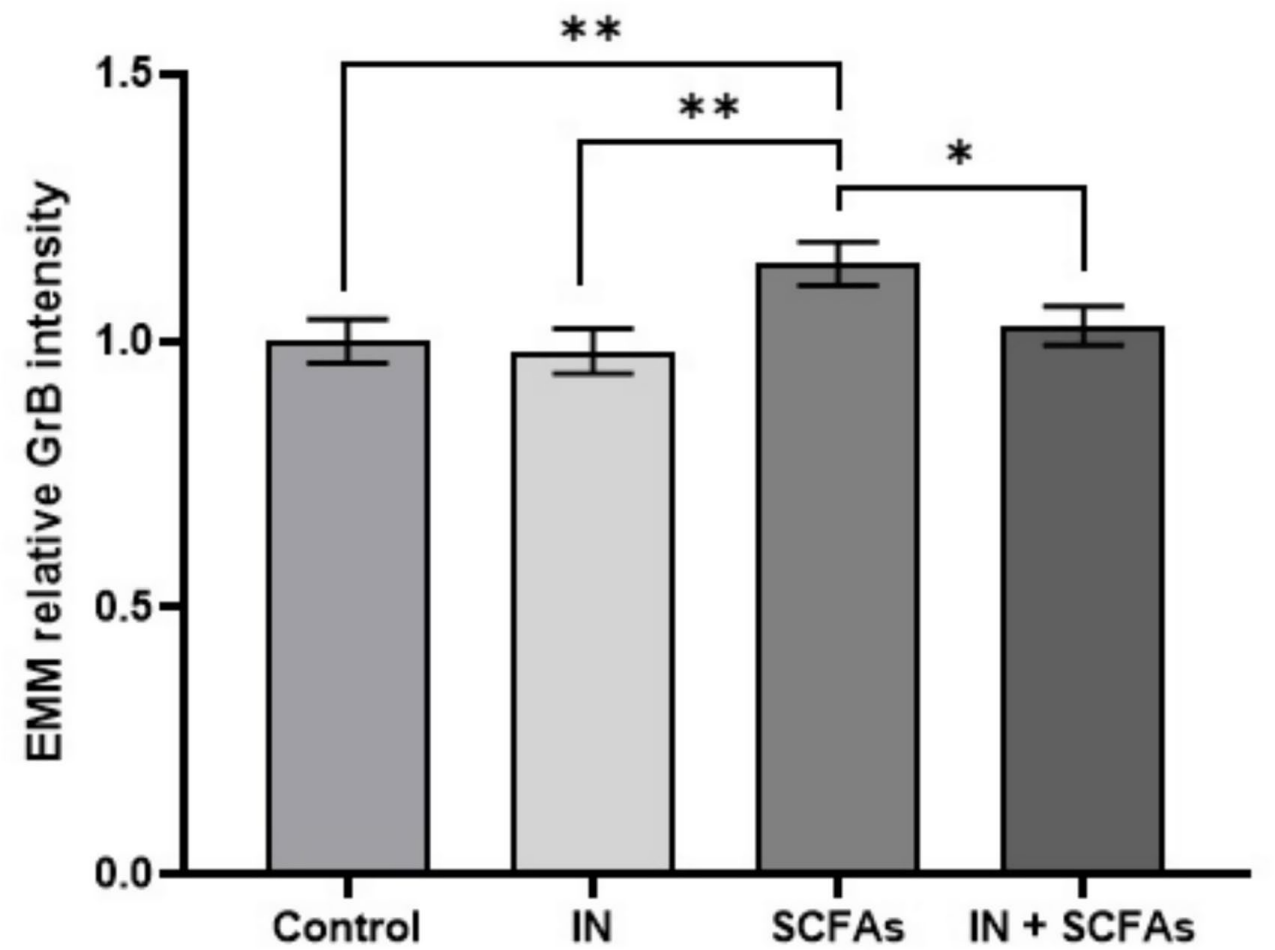
EMMs of relative GrB expression across experimental conditions (Error bars: SEM; *p < 0.05, and **p < 0.01).

To analyze the impact of experimental conditions on relative GrB expression, EMMs were calculated for each treatment group (Figure 7). The overall effect of treatment conditions was significant (*p* = 0.03). The mean EMMs ranged from 0.981 ± 0.042 (95% CI: 0.897–1.064) under IN condition to 1.145 ± 0.041 (95% CI: 1.063–1.228) under SCFA treatment. Control samples showed a mean of 1.000 ± 0.041 (95% CI: 0.919–1.081), while the combination of IN and SCFAs showed an intermediate value of 1.029 ± 0.036 (95% CI: 0.957–1.102). Pairwise comparisons indicated that SCFA treatment differed significantly from IN alone (*p* = 0.007) and from the combination of IN and SCFAs (*p* = 0.039). The difference between SCFA and control was also significant (*p* = 0.015).

The interaction between patient tissue characteristics and treatment condition also significantly influenced the relative GrB intensity (*p* < 0.001), indicating that the effects of IN, SCFAs, and their combination were patient-dependent. EMMs for each patient across conditions are presented in Table 5. While the GrB intensity in the control condition was normalized to 1.0, the response to the different conditions varied widely. In some patients tissue samples, IN or the combined IN and SCFA treatment led to a reduction in GrB intensity, whereas in others, the same conditions led to an increase. For instance, several individuals showed a decrease under combined treatment, whereas a subset of patients showed an increase, particularly under SCFAs alone. Between these differences, other patient tissue cultures showed relatively stable responses, with only slight changes regardless of treatment. These observations underscore the heterogeneity of patient responses and suggest that both the type of intervention and individual patient characteristics shape GrB outcomes.

**Table 5:** Estimated marginal means of relative GrB intensity for each patient across all treatment conditions.

Patient	Condition	EMM	SEM	95% Confidence interval	
				Lower bound	Upper bound
1	Control	1.000	0.108	0.784	1.216
	IN	0.432	0.108	0.216	0.649
	SCFAs	0.542	0.133	0.277	0.807
	IN + SCFAs	0.448	0.108	0.231	0.664
2	Control	1.000	0.108	0.784	1.216
	IN	1.631	0.133	1.366	1.896
	SCFAs	1.213	0.094	1.026	1.400
	IN + SCFAs	1.206	0.108	0.990	1.423
3	Control	1.000	0.133	0.735	1.265
	IN	0.975	0.108	0.758	1.191
	SCFAs	1.077	0.108	0.860	1.293
	IN + SCFAs	1.032	0.108	0.815	1.248
4	Control				
	IN				
	SCFAs	1.490	0.187	1.115	1.864
	IN + SCFAs	2.590	0.133	2.325	2.855
5	Control	1.000	0.133	0.735	1.265
	IN	0.795	0.133	0.530	1.060
	SCFAs	1.480	0.108	1.264	1.697
	IN + SCFAs	0.738	0.108	0.522	0.954
6	Control	1.000	0.108	0.784	1.216
	IN	0.749	0.108	0.533	0.966
	SCFAs	0.786	0.108	0.569	1.002
	IN + SCFAs	0.609	0.084	0.442	0.777
7	Control	1.000	0.108	0.784	1.216
	IN	1.078	0.108	0.861	1.294
	SCFAs	0.756	0.108	0.540	0.972
	IN + SCFAs	0.804	0.108	0.588	1.021
8	Control	1.000	0.108	0.784	1.216
	IN	1.009	0.133	0.744	1.274
	SCFAs	1.491	0.108	1.275	1.708
	IN + SCFAs	0.819	0.108	0.603	1.035
9	Control	1.000	0.108	0.784	1.216
	IN	1.177	0.108	0.961	1.393
	SCFAs	1.471	0.133	1.206	1.736
	IN + SCFAs	1.016	0.108	0.799	1.232

Values represent mean GrB intensity ± SEM with corresponding 95% confidence intervals.

### 3.3 Inflammatory markers within the supernatant

**Figure 8:**
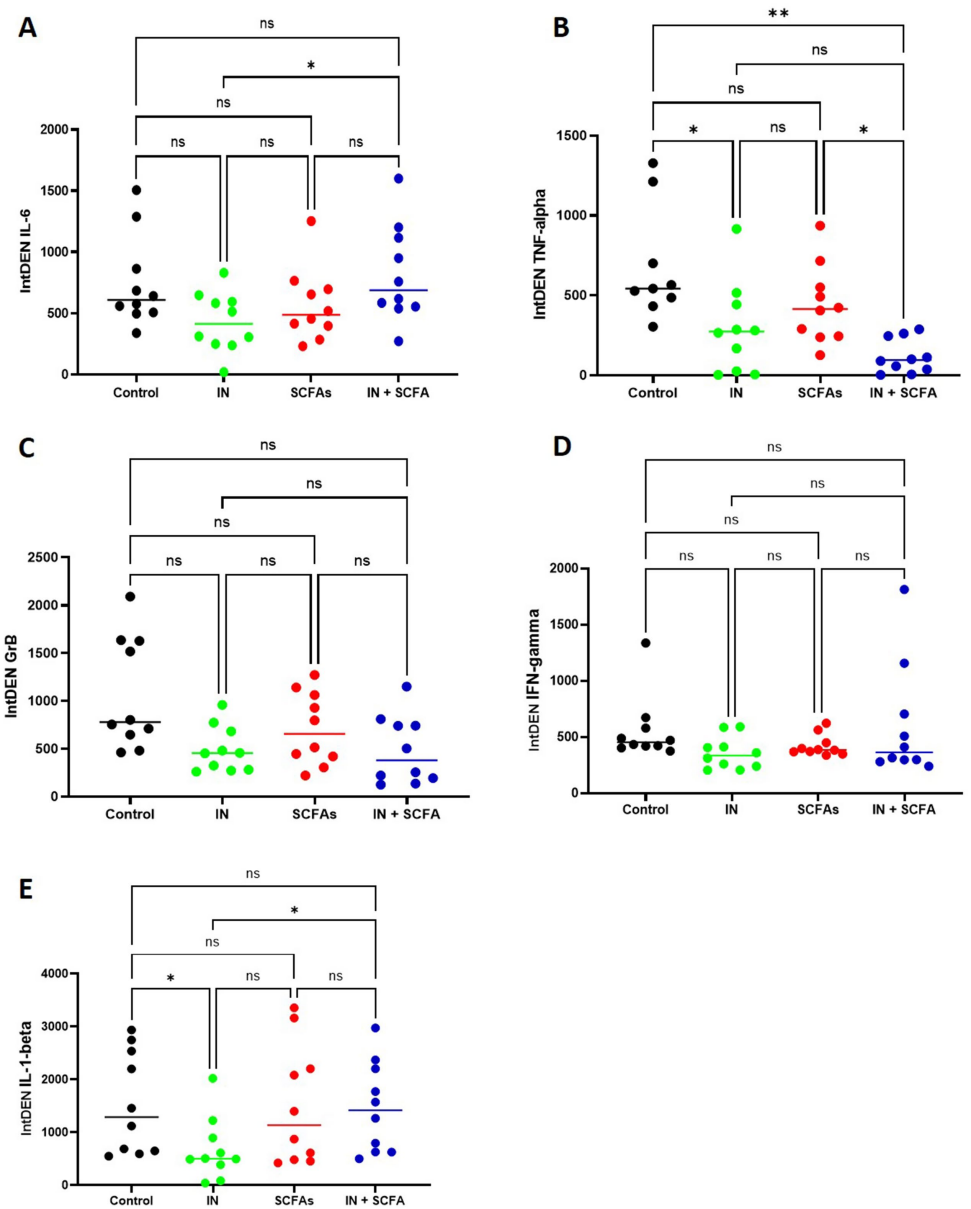
Pairwise comparison of inflammatory marker levels across treatment conditions. Relative integrated densities of IL-1β, IL-6, TNFα, GrB, and IFN-*γ* were measured in the supernatants of SC under control, IN, SCFAs, and combined IN + SCFA conditions. **(A)** Relative integrated densities of IL-6. **(B)** Relative integrated densities of TNFα. **(C)** Relative integrated densities of GrB. **(D)** Relative integrated densities of IFN-γ. **(E)** Relative integrated densities of IL-1β (Error bars: SEM; ns, not significant; *p < 0.05, and **p < 0.01).

To further assess the effects of the different treatments on immune activity, supernatants of SC were analyzed for inflammatory markers, including IL-1β, IL-6, TNFα, GrB and IFN-*γ* (Figure 8). Integrated density of IL-6 in the supernatant varied across treatment conditions, but overall differences were modest. Mean IL-6 intensity was 745.5 ± 372.5 for control, 428.5 ± 243.3 for IN, 566.3 ± 296.9 for SCFAs, and 818.8 ± 395.5 for the combined IN + SCFA condition. Pairwise comparisons indicated that most differences were not statistically significant. The only exception was a significant reduction in IL-6 levels for IN compared with the combined IN + SCFA treatment (mean difference −390.3; 95% CI − 753.8 to −26.8; *p* = 0.0354). All other comparisons, including control versus any treatment and SCFAs versus IN + SCFA, did not reach significance (Figure 8A). This was different for TNFα. Relative TNFα levels in the supernatant showed more pronounced differences across treatment conditions compared with IL-6. Mean intensity was 677.8 ± 117.8 for control, 290.7 ± 89.2 for IN, 442.2 ± 77.3 for SCFAs, and 119.9 ± 33.9 for the combined IN + SCFA treatment. Pairwise comparisons revealed significant changes in several conditions. Compared to control, TNFα levels were reduced under IN (mean difference −456.6; 95% CI 74.5–838.6; *p* = 0.0210) and relative to control lower in the combined IN + SCFA treatment (−557.1; 95% CI 176.5–937.7; *p* = 0.0068). Additionally, the combined treatment also showed a significant decrease compared to SCFAs alone (−322.3; 95% CI 44.6–600.0; *p* = 0.0236). All other comparisons were not statistically significant (Figure 8B). Similar to IL-6, relative GrB levels in the supernatant varied across conditions, but pairwise comparison did not show statistical significance. Mean intensities were 1,073 ± 583.7 for control, 494.7 ± 238.1 for IN, 710.8 ± 376.2 for SCFAs, and 487.6 ± 355.5 for the combined IN + SCFA treatment. Therefore, these results suggest a stable GrB response across treatments (Figure 8C). Relative IFN-*γ* levels in the supernatant showed some variation across treatment conditions, thus no significant difference was observed between the conditions. Mean intensities were 560.1 ± 287.4 for control, 357.5 ± 142.8 for IN, 422.8 ± 95.2 for SCFAs, and 602.1 ± 509.1 for the combined IN + SCFA condition. Similar to GrB, results indicate a generally stable IFN-γ response across treatments (Figure 8D). On the contrary, relative IL-1*β* levels in the supernatant varied across treatment conditions, with some significant differences observed. Mean intensities were 1,548 ± 966.3 for control, 676.6 ± 586.3 for IN, 1,505 ± 1,131 for SCFAs, and 1,471 ± 853.9 for the combined IN + SCFA condition. Pairwise comparisons showed that IL-1β levels were significantly reduced under IN compared with control (mean difference −871.5; 95% CI 50.5 to 1,692; *p* < 0.05) and significantly higher under IN + SCFA compared with IN alone (−794.5; 95% CI − 1,548 to −41.0; *p* < 0.05). All other comparisons were not statistically significant.

Overall, IL-6, GrB, and IFN-*γ* secretion remained largely stable across conditions, although the combination of IN + SCFAs appeared to counteract the small suppressive effect of IN alone on IL-6. In contrast, TNFα was particularly sensitive to IN and IN + SCFA treatment, indicating stronger modulation of this inflammatory marker. IL-1β secretion was also responsive to IN, with the combined IN + SCFA treatment maintaining higher levels compared with IN alone, highlighting differential cytokine-specific effects of these treatments.

### 3.4 Inflammatory markers within HNSCC slice cultures

**Figure 9:**
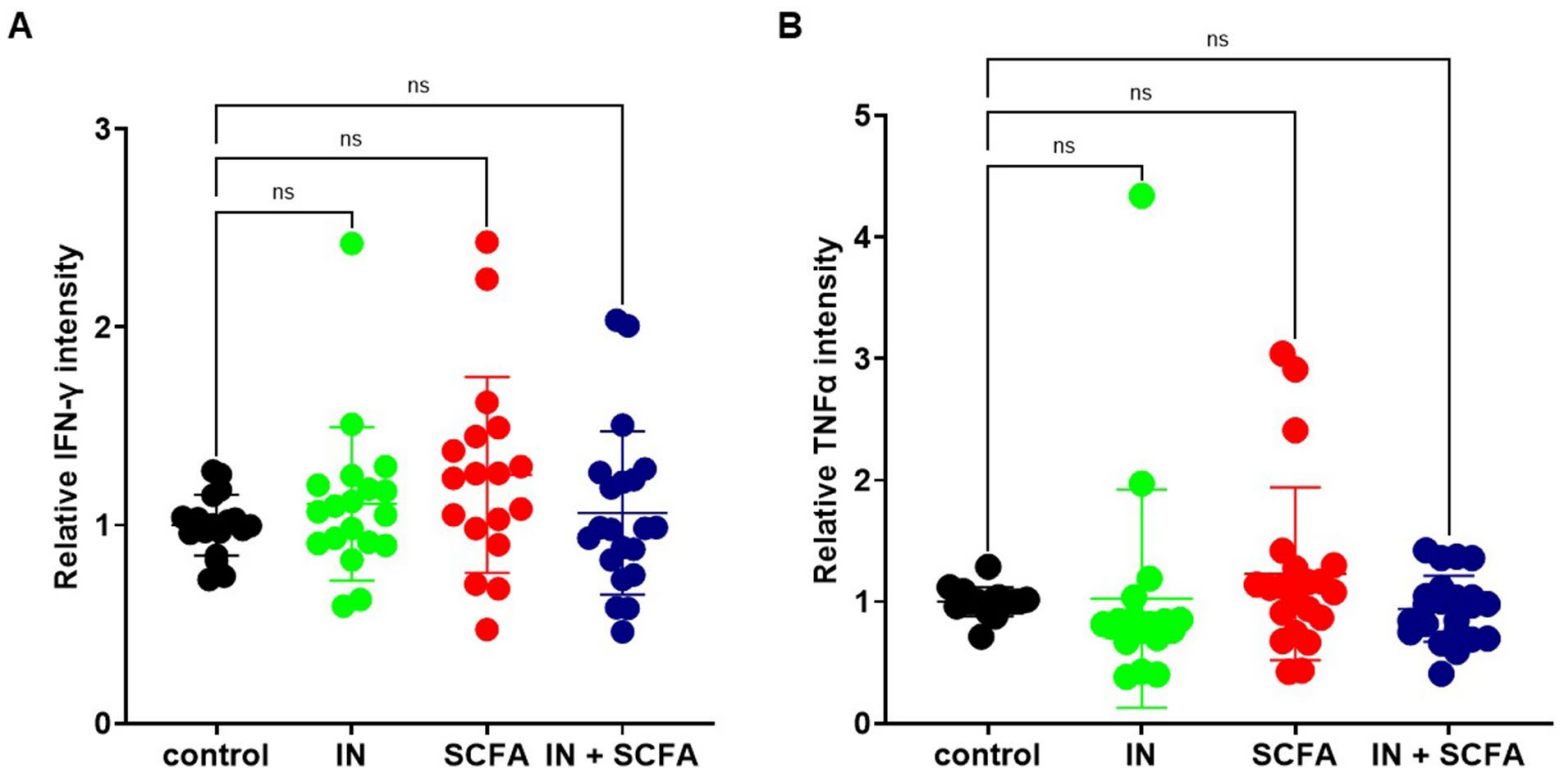
Comparison of relative intensities of TNFα and IFN-γ. **(A)** Relative IFN-γ intensity in control, IN, SCFA and IN+SCFAs. **(B)** Relative TNFα intensity in control, IN, SCFA and IN+SCFAs (ns, not significant).

To check if inflammatory markers were also present within the tissue of slice cultures, tissues were stained for TNFα, IL6, and IFN-γ. The relative intensities were quantified and compared across the four different experimental groups, as well as to the inflammatory marker levels measured in the culture supernatant. The mean relative IFN-γ intensity within SC showed only small variations between groups, with values remaining close to the control (control: 1.00 ± 0.036; IN: 1.11 ± 0.089; SCFA: 1.25 ± 0.117; IN + SCFA: 1.06 ± 0.090), suggesting that IN or SCFA treatment, alone or in combination, did not result in strong changes of IFN-*γ* within SC. Comparison of control versus IN revealed no significant change (*p* > 0.999), as well as control versus SCFA (*p* = 0.168) and control versus IN + SCFA (*p* > 0.999; Figure 9A).

Analysis of TNFα staining within SC showed similar relative intensities across the different treatment groups. Mean values remained close to control levels (control: 1.00 ± 0.030; IN: 1.03 ± 0.212; SCFA: 1.23 ± 0.155; IN + SCFA: 0.94 ± 0.058), indicating that neither IN nor SCFA caused significant changes in TNFα accumulation within the tissue. Moreover, comparing conditions did not reveal any significant changes (control vs. IN: *p* = 0.1467; control vs. SCFA: *p* > 0.999; control vs. IN + SCFA: *p* > 0.999; Figure 9B).

**Figure 10:**
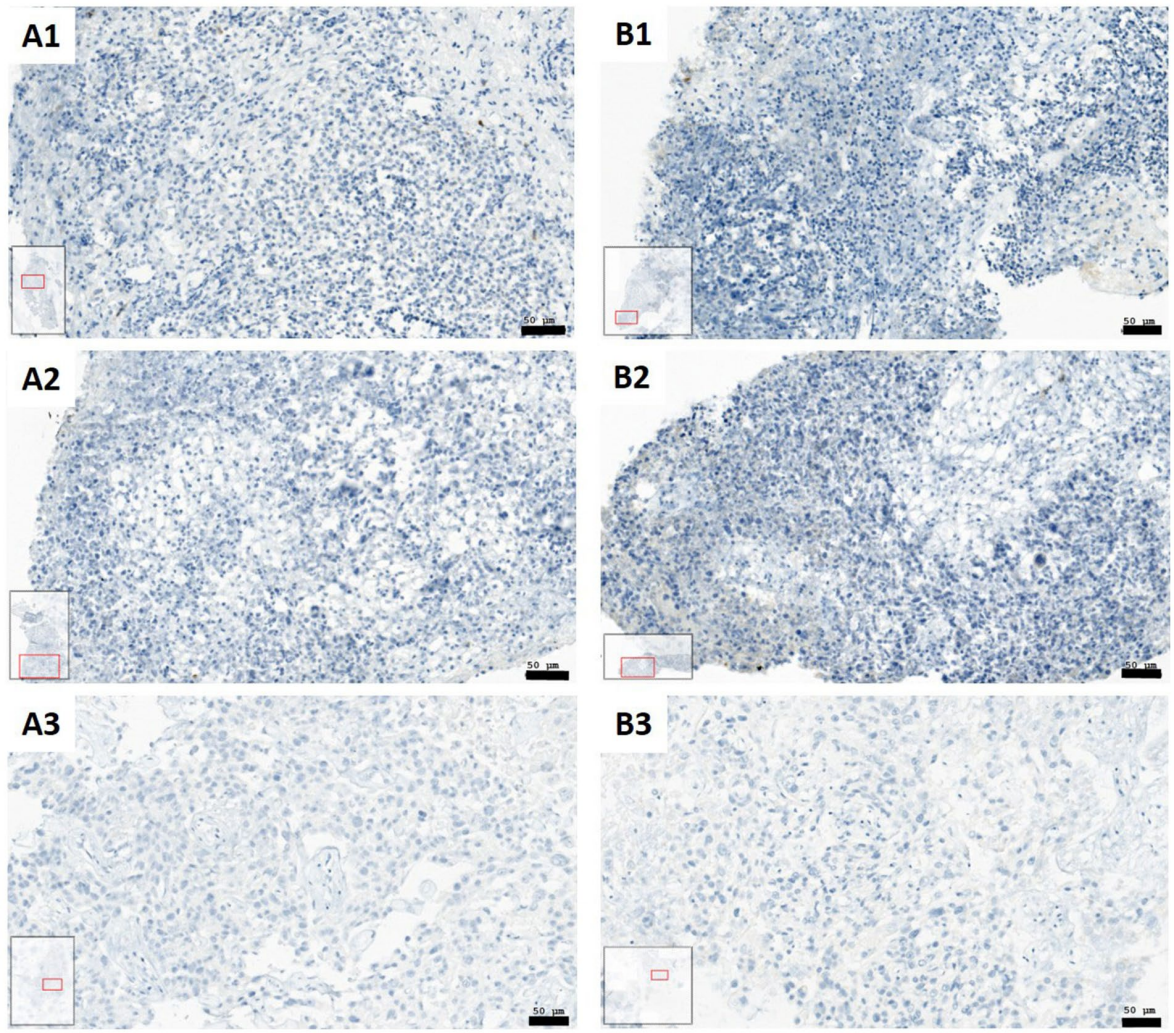
IL6 staining within SC (blue: IL6 negative cells; brown: IL6 positive cells). **(A1)** Control (Patient 8) with no brown (IL6-positive) cells; **(A2)** Control (patient 7) with no brown (IL6-positive) cells. **(A3)** Control (patient 1) with no brown (IL6-positive) cells; **(B1)** SCFA (patient 8) with almost no brown (IL6-positive) cells; sporadically one or two visible; **(B2)** SCFA (patient 7) with almost no brown (IL6-positive) cells; sporadically visible. **(B3)** SCFAs (patient 1) with no brown (IL6-positive) cells (Scale bars: 50 μm).

IL6 staining did not reveal detectable or only very weak positive signals across all examined tissue slices. Therefore, evaluation was performed by visual inspection and image documentation. Among the tested conditions, only SCFA treatment occasionally showed a slight increase in IL6 staining, though this effect was sporadic and remained extremely low overall (Figure 10).

Overall, analysis of TNFα, IL6, and IFN-γ in SC indicated that cytokine levels were similar across all treatment groups. TNFα and IFN-γ varied not significantly, while IL6 was nearly undetectable, with only occasional weak staining after SCFA treatment. Comparisons between tissue and supernatant showed no clear differences, indicating that inflammatory marker levels stayed stable in both.

## 4 Discussion

Patient-derived HNSCC SC were used to examine the effects of SCFAs and IN, alone or in combination, on apoptosis, cytotoxic activity, and inflammatory markers. IN, alone or combined with SCFAs, reduced CC3 intensity, indicating decreased apoptosis, whereas SCFAs alone increased apoptotic activity in certain patients´ samples. GrB IHC intensity showed no consistent modulation, and inflammatory cytokines within tissue and supernatant remained largely stable, except for selective changes in TNFα and IL-1. Responses varied considerably between patients, highlighting the heterogeneity of HNSCC tumors.

### 4.1 Effects of SCFAs and immunonutrition on apoptosis

Results demonstrate that SCFAs and IN exert opposing effects on apoptotic activity within HNSCC SC. SCFAs increased CC3 intensity compared with control, indicating a pro-apoptotic effect, whereas IN alone or in combination with SCFAs seemed to reduce CC3 activity. These findings are consistent with previous studies showing that SCFAs, particularly butyrate, can induce apoptosis through multiple mechanisms, like promotion of histone acetylation and modulation of mitochondrial pathways, which can lead to the activation of the intrinsic apoptotic cascade in cancer cells (40). Moreover, SCFAs have been shown to influence ferroptosis sensitivity through FFAR2-mTOR signaling, highlighting their capacity to modulate cell death via metabolic and epigenetic pathways (41). In a similar way, SCFAs can also trigger autophagy, which helps cells adapt to stress and may slow down mitochondria-related cell death, suggesting that their influence on tumor survival can depend on the specific context (42). Moreover, ex vivo studies in non-small cell lung cancer have also shown that SCFAs can cause tumor cell death and affect CD4^+^ T cells, highlighting their possible immunometabolic role (43). Overall, these studies indicate a possible pro-apoptotic effect of SCFAs similar to what was observed in HNSCC SC, while also suggesting that the response may depend on tumor type and cellular environment (40, 44).

On the other hand, IN containing glutamine, arginine and omega-3 fatty acids are known for their role in supporting cell survival and reducing oxidative stress (45, 46). Especially, arginine and lysine have been associated in the modulation of apoptotic and metabolic pathways in tumor cells, which may explain the reduced CC3 observed under IN conditions (45). Clinically, perioperative immunonutrition has been associated with improved outcomes in HNSCC patients, including lower postoperative complications and preservation of tissue integrity (33, 47). The decrease in apoptosis under IN conditions is usually indicated as cells better survive and that IN has anti-inflammatory effects. At the same time, reduction of apoptosis, also in terms of tumor cells apoptosis might have a tumor-supporting potential. Moreover, the observed heterogeneity across patients highlights the complexity of treatment responses in HNSCC. Some tumors displayed higher sensitivity to SCFA exposure with strong induction of apoptosis, while others showed minimal changes. This inter-patient variability may reflect differences in tumor genetics, metabolic state, and local immune composition, which is consistent with previous observations that SCFAs and immunonutritional factors act in a context-dependent manner (42, 44, 45). These results highlight that patient-specific factors could influence responses to metabolic and nutritional interventions, indicating that the effects of such approaches may vary between individuals. Granzyme B IHC, supernatant levels and cytotoxic activity.

Analysis of GrB intensity in SC did not show consistent changes across the treatment conditions. SCFAs were associated with slightly higher levels compared with IN or the combined IN + SCFA treatment, suggesting that they may increase cytotoxic potential based on GrB-producing cells as CD8^+^ T cells, NK cells in certain cases. This aligns with other studies showing that butyrate can increase the expression of cytotoxic molecules in CD8^+^ T cells and improve antitumor responses in immunotherapy models (48, 49). SCFA-induced modulation of GrB has also been observed in IL-10-producing Th1 cells, further indicating that SCFAs can influence cytotoxic potential (20). However, some patients ‘samples showed increased GrB intensity under SCFA treatment, while others had minimal changes or even reductions. This variability may be due to differences in tumor-infiltrating lymphocyte populations, immune checkpoint expression, or the metabolic state of each tumor, which supports the context-dependent effects of SCFAs on T cell function (22, 50). In addition, gut microbiota composition and SCFAs have also been linked to differences in antitumor immune responses, which could affect the efficacy of immunotherapy (50). These factors may help to explain why the cytotoxic effects of SCFAs were not uniform across all patients.

The limitation of research material did not allow more detailed immune phenotyping which is obvious limitation of this study.

In contrast, IN was associated with a slightly reduced GrB intensity in many samples, which may be due to the immunomodulatory effects of omega-3 fatty acids and amino acids. These nutrients are known to reduce inflammatory responses and shift T cell activity toward regulatory or anti-inflammatory phenotypes (51). Clinical studies have similarly reported that perioperative immunonutrition in HNSCC patients reduces inflammatory complications and supports the immune system (33, 47). The results indicate that SCFAs might boost cytotoxic T cell activity, while IN may promote more regulatory effects, depending on the TME and the patient’s immune profile.

### 4.2 Cytokines in the supernatant and within tumor tissues

Analysis of cytokines in the culture supernatant revealed a mediator-specific response to SCFAs and IN. IL-6, IFN-*γ*, and GrB levels remained largely stable across all treatment conditions, whereas TNFα and IL-1β demonstrated more pronounced reductions under IN and the combined IN + SCFA conditions. These observations align with clinical studies reporting that immunonutrition can modulate inflammatory pathways and reduce systemic inflammatory markers in cancer patients undergoing surgery or chemoradiotherapy (31, 52, 53, 54, 55). For instance, perioperative IN has been shown to decrease circulating IL-6 and TNFα while enhancing antioxidant defences in leukocytes, supporting the notion that IN exerts both anti-inflammatory and immunomodulatory effects (54, 55). Similarly, omega-3 fatty acids and specific amino acids present in IN are known to influence cytokine production by dampening pro-inflammatory responses and promoting regulatory immune functions (31).

The relatively stable levels of IL-6, IFN-*γ*, and GrB under SCFA treatment illustrate the dual role of SCFAs in immune modulation. While systemic studies often report SCFA-mediated suppression of pro-inflammatory cytokines, their effects can vary depending on tissue context, cell type, and local metabolic conditions (13, 56, 57). SCFAs have been shown to enhance regulatory T cell function and modulate effector T cell activity, sometimes maintaining or even increasing cytokine secretion in certain microenvironments (13, 57). This context-dependent behavior could explain why SCFAs did not consistently reduce cytokine levels in the HNSCC slice cultures, but in some cases appeared to preserve baseline inflammatory signaling.

The immunohistochemical assessment of tissue slices supported the supernatant findings. TNFα and IFN-γ staining did not show significant changes across treatment conditions, and IL-6 was nearly undetectable except for sporadic weak signals in SCFA-treated samples. This suggests that, within the preserved tissue architecture of the slice cultures, cytokine levels may remain buffered against external interventions over the four-day culture period. It is possible that SCFA- or IN-induced cytokine production occurs rapidly and transiently after treatment initiation, and the single end-point measurement at day four may have missed early spikes in cytokine release (12). Short-lived inflammatory changes might have been present in the cultured tissue samples, which could alter interpretation of the largely “stable” cytokine profiles observed. In fact, cytokines might be secreted only transiently, these still can be translated to long-term gene expression changes (58). For example upon T cell activation, IFNγ is produced for 3–10 h, but IFNγ – induced gene upregulations might be detected as peak of two days, which conditions are within our observation range. One such IFNγ-regulated gene was GrB (58). In case of both IFNγ and GrB we observed the above mentioned buffer effect. Indeed, short-lived inflammatory stimuli can significantly increase immune function and antigene presentation, which was seemingly not the case in our study. Especially repeated treatments might achieve memory effects (59).

Overall, these results indicate that IN exerts anti-inflammatory effects by reducing TNFα and IL-1β secretion, whereas SCFAs maintain cytokine levels without inducing substantial suppression. The differential patterns highlight cytokine- and treatment-specific effects and suggest that timing, local tissue context, and slice integrity are critical factors in interpreting ex vivo cytokine responses (12, 13, 57).

### 4.3 Implications for therapy in HNSCC

The different actions of SCFAs and IN on apoptosis suggest that metabolic inputs can shift the balance between cell death and survival in tumor tissue. The selective reduction of TNFα and IL-1β by IN further indicates potential anti-inflammatory benefits, which may contribute to reduced tissue stress but could also dampen immune-mediated tumor control. SCFAs, in contrast, appear more pro-apoptotic and potentially supportive of cytotoxic activity, though highly dependent on individual patient context.

The used concentration of SCFAs requires a further point of discussion. There is a contrast between the measured concentration of SCFAs in colon tissue and serum concentrations, which display rapid concentration decreases (60). We used concentrations that are comparable with treatments published (38), and also comparable with possible maximal concentrations in the human body considering the maximal tissue exposition (61).

These findings have implications for nutritional strategies in HNSCC. IN is commonly used in perioperative and supportive settings to improve patient outcomes, but its potential impact on tumor biology and immune responses remains under debate (62, 63). The results presented here suggest that IN may help maintain tissue integrity while reducing inflammation, but it might also attenuate apoptosis and cytotoxicity within the tumor. SCFAs, on the other hand, could represent a metabolic modulator capable of inducing apoptosis and enhancing effector activity in certain tumors. Therefore, future research should further investigate molecular mechanisms, integrating metabolic profiling and immune phenotyping to better predict patient-specific responses. In addition, extending culture periods or combining nutritional interventions with targeted therapies could clarify their relevance for clinical strategies.

### 4.4 Limitations and strengths

This study has several aspects that should be considered when interpreting the findings. The number of patient-derived samples was limited, which may restrict generalizability, but at the same time, the use of fresh human HNSCC tissue represents a major strength compared to cell line or animal models. Cytokine analysis was performed only at a single time point (day 4), which may have missed rapid, early changes in cytokine release. It is possible that SCFA or IN induced a fast initial immune switch that declined over time, leading to the overall low cytokine intensities detected. Nevertheless, the focus on a later time point allowed assessment of sustained rather than transient effects, which adds complementary information. Another consideration is the complexity and heterogeneity of HNSCC, which can influence immune responses across patients. Overall, while acknowledging limitations, this study shows the value of organotypic SC for investigating IN interventions in HNSCC and provides an important basis for future work with larger cohorts, additional time points, and expanded mechanistic analyses.

## 5 Conclusion

In conclusion, this study demonstrates that SCFAs and IN differentially modulate apoptosis, immune activity, and cytokine secretion in HNSCC SC. SCFAs tended to promote apoptosis and, in some cases, enhance cytotoxic activity, while IN reduced apoptotic activity and suppressed pro-inflammatory cytokines such as TNFα and IL-1. Cytokine profiles in both tissue and supernatant remained largely stable, with IL-6 showing almost no detectable staining. These findings suggest that nutritional interventions exert context-dependent effects on tumor biology, influenced by both treatment composition and patient-specific factors. Further research with larger cohorts, earlier time points, and integrative immunometabolic analyses will be necessary to clarify how such interventions may be used to optimize supportive care or even therapeutic strategies in HNSCC.

## Data Availability

The datasets presented in this study can be found in online repositories. The raw data and figures can be found in Zenodo: https://zenodo.org/records/17424808.
