# Supplementary material for: Immunomodulatory effects of short-chain fatty acids and immune-supporting nutrients on slice cultures of head and neck tumors
Source: Front Nutr. 2026 Mar 13;13:1731077. doi: 10.3389/fnut.2026.1731077 (PMC13021408; doi:10.3389/fnut.2026.1731077)
Supplement: Supplementary file 1 [file Table_1.DOCX]

***Supplementary Table 1.:*** *Estimated marginal means of relative GrB intensity for infiltration stages of CD45^+^ cells across treatment conditions. Values represent mean GrB intensity ± SEM with corresponding 95 % confidence intervals.*

| **Dependent Variable: GrB relative intensity** | | | | | |
| --- | --- | --- | --- | --- | --- |
| **CD45** | **Treatment** | **EMM** | **SEM** | **95% Confidence Interval** | |
|  |  |  |  | **Lower Bound** | **Upper Bound** |
| **immune desert** | **Control** | 1,000 | 0,125 | 0,751 | 1,249 |
|  | **IN** | 0,786 | 0,125 | 0,537 | 1,035 |
|  | **SCFAs** | 0,912 | 0,142 | 0,630 | 1,194 |
|  | **IN + SCFAs** | 0,676 | 0,113 | 0,451 | 0,901 |
| **immune exluded** | **Control** | 1,000 | 0,265 | 0,472 | 1,528 |
|  | **IN** | 0,975 | 0,217 | 0,544 | 1,406 |
|  | **SCFAs** | 1,077 | 0,217 | 0,646 | 1,508 |
|  | **IN + SCFAs** | 1,032 | 0,217 | 0,601 | 1,463 |
| **infiltrated** | **Control** | 1,000 | 0,113 | 0,775 | 1,225 |
|  | **IN** | 1,123 | 0,125 | 0,874 | 1,371 |
|  | **SCFAs** | 1,252 | 0,100 | 1,052 | 1,451 |
|  | **IN + SCFAs** | 1,135 | 0,100 | 0,935 | 1,334 |
